# Bias in the association between advanced maternal age and stillbirth using left truncated data

**DOI:** 10.1038/s41598-022-23719-3

**Published:** 2022-11-10

**Authors:** Jennifer Dunne, Gizachew A. Tessema, Amanuel T. Gebremedhin, Gavin Pereira

**Affiliations:** 1 Curtin School of Population Health, Curtin University, Bentley, WA Australia; 2 School of Public Health, University of Adelaide, Adelaide, SA Australia; 3 enAble Institute, Curtin University, Bentley, WA Australia; 4 Centre for Fertility and Health (CeFH), Norwegian Institute of Public Health, Oslo, Norway; 5 Curtin University, GPO Box U1987, Perth, WA 6845 Australia

**Keywords:** Reproductive disorders, Epidemiology

## Abstract

Restriction to analysis of births that survive past a specified gestational age (typically 20 weeks gestation) leads to biased exposure-outcome associations. This bias occurs when the cause of restriction (early pregnancy loss) is influenced by both the exposure and unmeasured factors that also affect the outcome. The aim of this study is to estimate the magnitude of bias resulting from left truncated data in the association between advanced maternal age and stillbirth. We simulated data for the causal pathway under a collider-stratification mechanism. Simulation parameters were based on an observed birth cohort from Western Australia and a range of plausible values for the prevalence of early pregnancy loss, unmeasured factor *U* and the odds ratios for the selection effects. Selection effects included the effects of maternal age on early pregnancy loss, *U* on early pregnancy loss, and *U* on stillbirth. We compared the simulation scenarios to the observed birth cohort that was truncated to pregnancies that survived beyond 20 gestational weeks. We found evidence of marginal downward bias, which was most prominent for women aged 40 + years. Overall, we conclude that the magnitude of bias due to left truncation is minimal in the association between advanced maternal age and stillbirth.

## Introduction

It is considered that women with advanced maternal age (> 35 years of age) have an increased risk of stillbirth^1^. However, the magnitude of this increased risk is unclear when using birth data that is restricted to pregnancies that survive beyond a specified gestational week^2^, as the exposure may impact selection into the study and thus mask the true observation of outcomes. In high-income settings, selection into a study is generally restricted to pregnancies that survive beyond 20 gestational weeks^3^, a time when pregnancy is considered clinically viable. Thus, the use of left truncated birth registries and cohort studies that recruit women during a specific period of pregnancy, will produce biased estimates in perinatal exposure-outcome associations. The mechanism that leads to these biased associations is collider stratification bias. This occurs as conditioning on a collider, a common effect of an exposure and an outcome, induces a correlation between the exposure and a confounder^4^. If the confounder also affects the outcome, conditioning on the collider leads to a specious association that is either strengthened or reversed between the exposure and outcome^5^. The most well-known example of collider-stratification bias in perinatal epidemiology is the birth-weight paradox^6^. In this example, stratifying on birth weight produces a cross-over of the birth-weight mortality curves, such that low birth weight babies with smoking mothers have a lower mortality rates than low birth weight babies with non-smoking mothers^7^. However, the collider-stratification mechanism that underpins bias resulting from left truncated data is more difficult to address analytically as selection is based on an attrition processes that we cannot observe in data, i.e. early pregnancy loss.

With estimates of 2500 early pregnancy losses per 10,000 implantations^8^, an extensive cohort attrition has already occurred prior to pregnancy being established due to spontaneous and induced abortion. The exact aetiology of spontaneous abortion remains unclear, although it is widely acknowledged that they result from interaction between hormonal, immunology, genetic and environmental factors^9–12^. Parental age is considered to be a strong risk factor for early pregnancy loss^11,13^, with the risk of early pregnancy loss slightly elevated in younger mothers before rising sharply in older mothers (≥ 35 years)^11^. The continuing trend of advanced maternal age and high rates of stillbirth in high-income settings have led many researchers to examine the association between the exposure of advanced maternal age and the outcome of stillbirth, defined as fetal death at 20 gestational weeks or more. Advancing maternal age (≥ 35 years) has been identified is an independent risk factor for stillbirth^1^, with the increased risk of stillbirth not accounted for by increased prevalence of other maternal comorbities^14^. In studies that use left truncated datasets (i.e. missing pregnancies prior to 20 gestational weeks), the differential impact of maternal age on early pregnancy loss will lead to biased estimates in the relationship between advanced maternal age and stillbirth. Whether the bias is of concern will depend on its magnitude and direction, which remain unclear. Because early pregnancy losses are unobserved, simulations are a useful tool for exploring the influence of bias resulting from such left truncated data on the effects of exposure prior to pregnancy on birth outcomes^15^. In this simulation study, we aimed to quantify the influence of bias due to left truncation and selection in utero on the association between the exposure of advancing maternal age and the risk of stillbirth in a population representative of high-income settings.

## Methods

The motivation for this study was to quantify the influence of bias due to left truncated birth data in the association between advanced maternal age at conception and stillbirth. Using data from the Midwives Notification Systems (MNS) in Western Australia, we compared effect estimates with those from simulated models in which we adjusted for the influence of selection bias under a range of plausible scenarios. For this study, we considered early pregnancy loss as fetal death prior to 20 gestational weeks; and stillbirth when fetal deaths occurred at 20 gestational weeks or later^16^.

### Observed cohort

The observed cohort consisted of women who had a singleton birth in Western Australia between 1998 and 2015 (births = 483,466), derived from the MNS^16^. This de-identified and validated dataset contains all births in Western Australia with either a gestational length ≥ 20 gestational weeks or a birth weight > 400 g^16^. We cross-referenced the MNS with Death Registrations obtained from the WA Registry of Births, Deaths and Marriages using a linkage key provided by the Data Linkage Branch of the WA Department of Health^17^. Hospitalisation records were identified from the Hospital Morbidity Data Collection for WA using the Australian Modification of International Classification of Diseases (ICD-9:779.9; ICD-10:P45 and P96.9) coded diagnostic information for stillbirth^18^. We categorised maternal age into five- year age groups (20–24, 25–29, 30–34, 35–39 and 40 + years). As the primary interest of this study is the biological impact of advancing age on stillbirth, women younger than 20 years were excluded in both the observed cohort and simulation study.

### Bias structure

**Figure 1:**
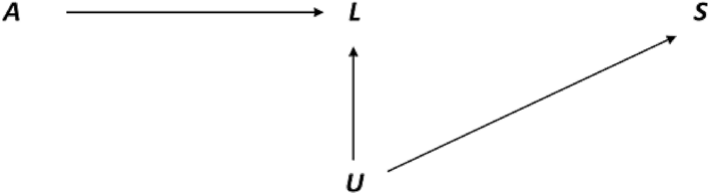
Directed acyclic graph (DAG) of the structure of collider-stratification bias. The exposure maternal age *A* affects early pregnancy loss *L*, which is also affected by the independent risk factor *U*, inducing a back-door pathway between exposure *A* and the outcome of stillbirth *S*.

The causal diagram (Fig. 1) illustrates the bias resulting from restriction to births that survive past 20 gestational weeks. Here, the exposure *A* (maternal age, a proxy for aging) affects early pregnancy loss *L*. An unmeasured confounder *U* is causally associated with increased risk of pregnancy loss *L* and the outcome of stillbirth *S*. Both the exposure *A* and the unmeasured confounder *U* independently affect early pregnancy loss *L,* which is a collider. Thus, by excluding pregnancies that end in loss prior to 20 weeks gestation (*L* = 1), or conditioning on *L*, a back-door pathway is opened from maternal age to stillbirth through the pregnancy loss *L* and the unknown confounder *U*. This bias is commonly known as collider-stratification bias. An assumption implicit in the causal diagram is that maternal age causes early pregnancy loss, however, after attaining a gestational length close to viability (here 20 gestational weeks), maternal age has no direct influence on risk of stillbirth.

### Simulation

To quantify the influence of the collider-stratification bias on the association between advanced maternal age and stillbirth, we simulated a population of 500,000 conceptions which is approximately the number of births in the observed cohort. We generated data for the maternal age exposure *A*, unmeasured confounder *U*, early pregnancy loss *L* and the outcome of stillbirth *S*. Maternal age variable *A* was normally distributed, with the mean and standard deviation derived from the Gaussian distribution of age in the observed cohort. As per the observed cohort, we categorised maternal age into five-year age groups (20–24; 25–29; 30–34; 35–39; 40–45) and excluded mothers younger than 20 years. The early pregnancy loss variable *L*, the unmeasured variable *U* and the stillbirth variable *S* were binary variables. The prevalence of *L* (π_*L*_) was set to 12.8%^11^, 20%^19^ and 30%^20^ to reflect a realistic range of early pregnancy loss as reported in high-income settings. The baseline prevalence of *S* was set to 0.7% to reflect the incidence of stillbirth in the observed cohort. We set the prevalence of *U* (π_*U*_) to 0.15, 0.30 and 0.50, to reflect a range of plausible scenarios.

**Figure 2:**
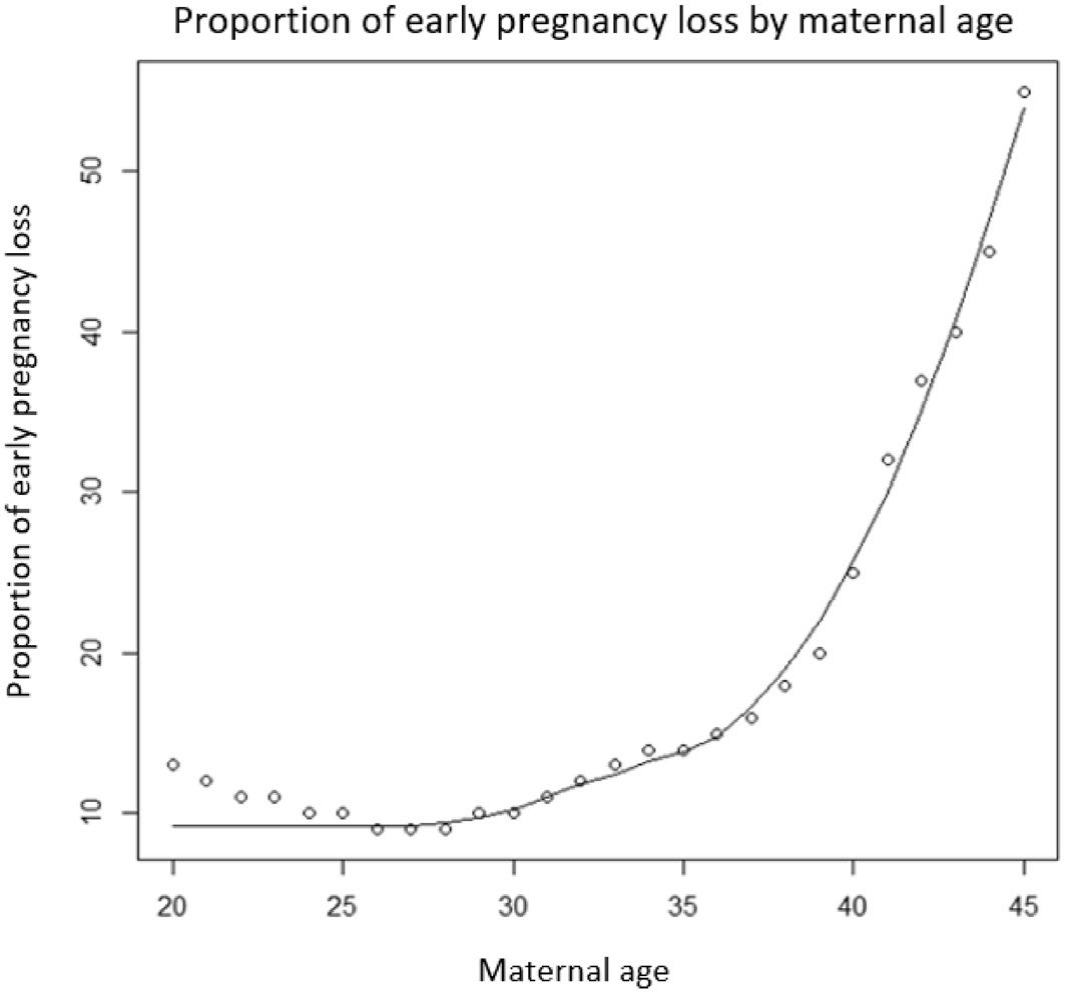
Risk of early pregnancy loss according to maternal age with locally weighted scatterplot smoothing curve.

The overall causal pathway [*A→L←U→S*] that represents the collider-stratification bias was broken down to smaller pathways [*A→L*, *U→L*, *U→S*], which we deemed ‘selection effects’. All selection effects were modelled in terms of odds ratios (ORs) so that simulation probabilities were bounded between 0 and 1. For the selection effect *A→L,* we assigned each individual an underlying risk of early pregnancy loss based on their biological age at conception, which was drawn from a Bernoulli model based on results from a 2019 Norwegian study^11^ of the effects of maternal age on early pregnancy loss. The Norwegian study^11^ reported the lowest risk of miscarriage among women aged 25–29 (9.8%), with an absolute lowest risk at age 27 (9.5%) and the highest risk at age 45 (53.6%). As we were unable to ascertain the increasing risk of early pregnancy loss for women aged older than 45 years, we limited our simulation study to women aged between 20 and 45 years. In our Bernoulli model we used non-parametric regression to capture the nonlinearity of the association between the exposure and early pregnancy loss using LOESS (locally weighted scatterplot smoothing)^21^ (Fig. 2).

The probability of early pregnancy loss for each conception *i* (assuming a monotonic risk by maternal age) was estimated using the equation below:

$$P\left( {L_{i} } \right)\, = \,\frac{{\exp \left( {\beta_{0} \, + \,\beta_{1} A_{i} \, + \,\beta_{2} U_{i} } \right)}}{{1\, + \,\exp \left( {\beta_{0} \, + \,\beta_{1} A_{i} \, + \,\beta_{2} U_{i} } \right)}}.$$

Selection effects for *U→L* and *U→S* were set to an equal OR from a range of 1.5, 2.0, 2.5 and 3.0. To isolate the bias mechanism we firstly assumed a true null effect of maternal age on stillbirth (i.e. there is no direct causal effect of *A→S*). We further considered a scenario in which there was an interaction between the unmeasured confounder *U* and maternal age *A* on early pregnancy loss *L* in conjunction with the collider-stratification mechanism. Often called depletion of susceptibles, the interaction of A*U increases the prevalence of early pregnancy loss for those that are exposed to both the exposure *A* and *U* (Fig. S1). Selection effects for A*U were set to an equal OR as with the selection effects for *U—*> *L* and *U→S,* with a range set to 1.5, 2.0, 2.5 and 3.0. To enable a direct comparison with the observed cohort, we then considered a third scenario in which we assumed a true effect of maternal age on stillbirth *A→S* (Fig. S2). Here each individual was assigned a probability of stillbirth drawn from a Bernoulli model based on the risk of stillbirth from their biological age of the observed cohort at conception (Fig. S3). To capture the nonlinearity of this direct association between the exposure maternal age *A* and the outcome of stillbirth *S* we conducted non-parametric regression with LOESS^21^.

### Analysis

We estimated the OR for the association between the exposure and outcome in the observed cohort and the simulated populations. We performed logistic regression of stillbirth with maternal age as the exposure to obtain the OR, which approximates the risk ratio because the outcome of stillbirth is rare in Western Australia^22^. We exponentiated the mean of the point estimates obtained from 100 iterations for each scenario to obtain OR_*AS|L*=*0*_, which represents the OR for the effect of *A* on *S* for pregnancies in which early pregnancy loss did not occur (*L* = 0). We then derived the percentile-based 95% simulation intervals (SI) of the OR mean using 500 bootstrap replications.

We initially examined the collider-stratification bias under a range of plausible assumptions by varying the selection effects (OR_*UL*_ and OR_*US*_) and the prevalence of both *L* and *U* as described above. In the first scenario, the simulation is conducted under the null hypothesis of no association between advancing maternal age *A* with the exposure of stillbirth *S*. In the second scenario we simulated a collider-stratification mechanism with an association between the exposure *A* and the unmeasured confounder *U*. As in the first scenario, we conducted the simulation under a hypothesis of no association between advancing maternal age *A* and stillbirth *S*. In both scenario one and scenario two we assumed that there is no causal effect, and therefore the value of OR_*AS|L*=*0*_ was set to 1. Consequently, we interpreted the results such that the greater the departure of OR_*AS|L*=*0*_ from 1 the greater the magnitude of the bias.

For the third scenario in which we assumed a true effect of *A→S,* we were able to undertake a direct comparison with the observed cohort. For OR_*AS|L*=*0*_ in this scenario, we simulated collider-stratification mechanism without an association between exposure *A* and the unmeasured confounder *U* and assumed a true effect of the exposure *A* on the outcome stillbirth *S*. Here the greater difference between OR_*AS|L*=*0*_ and OR_*AS*_ (the observed cohort without the simulated bias), the greater the magnitude of bias. Furthermore, to eliminate possible model misspecification due to the categorisation of maternal age, we undertook a sensitivity analysis in which we simulated the true null association between the exposure maternal age *A* and the outcome of stillbirth *S* with input parameters π_*L*_ = 0.20, π_*U*_ = 0.15, OR_*UL*_ = 1.5, OR_*US*_ = 1.5 for each whole year of maternal age (Fig. S5). All data analyses and simulations were conducted using R v4.0.5^23^.

### Ethical approval

This study was conducted in accordance with the principles of the Declaration of Helsinki. Ethical approval for this study was obtained from the Human Research Ethics Committee, Department of Health, Western Australia (HREC approval 2016/51) with a waiver of participants' informed consent, particularly due to the implausibility of obtaining retrospective consent for de-identified secondary data.

## Results

**Figure 3:**
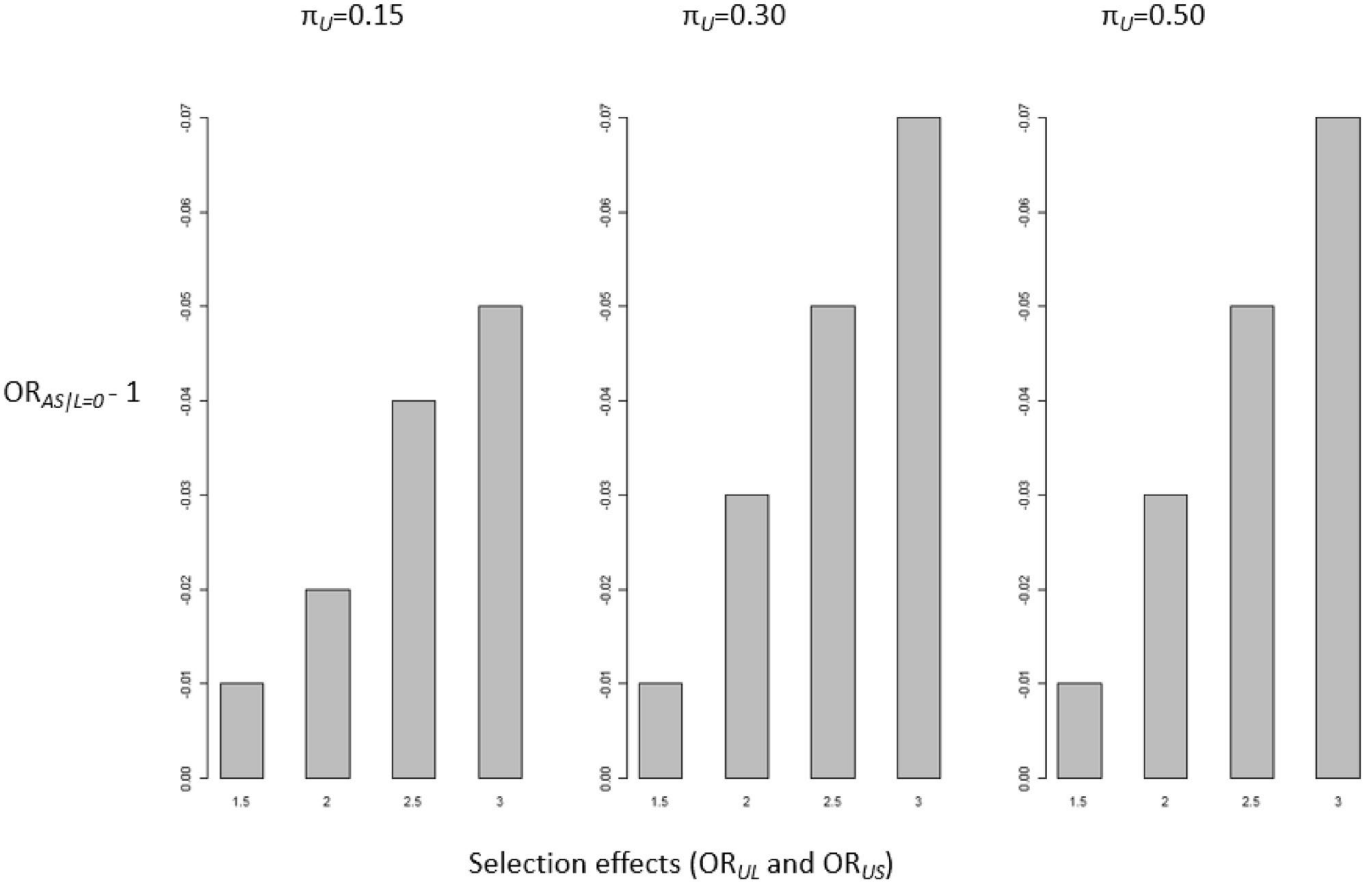
Collider-stratification bias of OR_*AS|L*=*0*_ − 1 under the true null effect of maternal age on stillbirth for women aged 40 + years, where the bias represents the departure from the null. Average odds ratio (OR_*AS|L*=*0*_) with π_L=_ 0.20 and with varying input parameters for π_*U*_ (0.15, 0.30, 0.50) and the selection effects OR_*UL*_ and OR_*US*_ (1.5, 2.0, 2.5, 3.0). Each scenario was iterated 100 times.

Overall, the bias was minimal under a true null association between the exposure maternal age *A* and the outcome of stillbirth *S*. In scenario one, we considered a collider-stratification bias where the exposure maternal age *A* and the unmeasured confounder *U* independently effected early pregnancy loss (Table S1). Here the magnitude of bias was generally weak for women aged 35–39 years, with departure from 1 not evidenced until the selection effects (OR_*UL*_ and OR_*US*_) were set to a minimum of 2.5 and regardless of the values of π_*L*_ and π_*U*_. For example, the OR_*AS|L*=*0*_ for women aged 35–39 years was 0.98 (SI 0.97 to 0.99) with input parameters of π_*L*_ = 0.128, π_*U*_ = 0.30, OR_*UL*_ = 3.0, OR_*US*_ = 3.0. For women aged 40 + years there was evidence of increasing bias when the magnitudes of the selection effects increased (OR_*UL*_ and OR_*US*_) regardless of the values of π_*L*_ and π_*U*_ (Fig. 3). The largest departure from the null for women aged 40 + years was evident with input parameters of π_*L*_ = 0.128, π_*U*_ = 0.30, OR_*UL*_ = 3.0, OR_*US*_ = 3.0 (OR_*AS|L*=*0*_ 0.92 SI 0.90 to 0.94).

**Figure 4:**
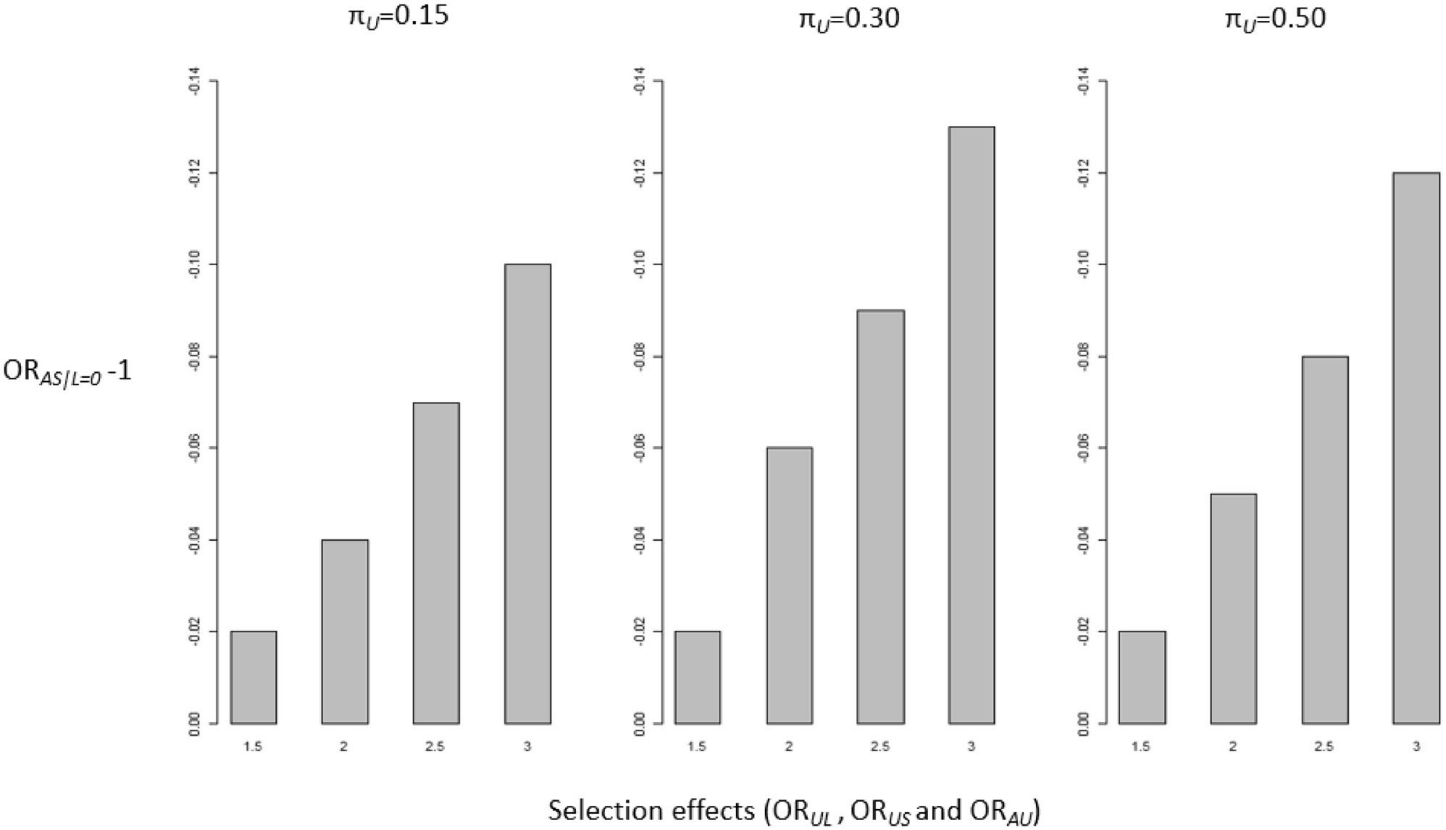
Collider-stratification bias of OR_*AS|L*=*0*_ − 1 under the true null effect of maternal age on stillbirth for women aged 40 + years with an interaction between exposure *A* and the unmeasured confounder *U*, where the bias represents the departure from the null*.* Average odds ratio (OR_*AS|L*=*0*_) with π_L=_ 0.30 and with varying input parameters for π_*U*_ (0.15, 0.30, 0.50) and the selection effects (OR_*UL*_, OR_*US,*_ OR_*AU*_). Each scenario was iterated 100 times.

In the second scenario, when we considered the collider-stratification mechanism with an interaction between the exposure *A* and the unmeasured confounder *U*, we found a greater departure from the null for women aged 40 + compared to scenario one. In this scenario, we also found that the magnitude of the bias increased with increasing values of π_*L*_ and π_*U*_ (Fig. 4). The strongest evidence of bias was evident in women aged 40 + years with π_*L*_ = 0.30, π_*U*_ = 0.30, OR_*UL*_ = 3.0, OR_*US*_ = 3.0 (OR 0.87 SI 0.84 to 0.89) (Table S2). For women aged 35–39 years, there no evidence of bias when the selection effects (OR_*UL*_, OR_*US,*_ OR_*AU*_) were set to 1.5 and 2.0, regardless of the values of π_*L*_ and π_*U.*_ The greatest departure from the null was evidenced (OR_*AS|L*=*0*_ 0.98 SI 0.97 to 0.99) when π_*L*_ = 0.30, OR_*UL*_ = 3.0, OR_*US*_ = 3.0, OR_*AU*_ = 3.0 and π_*U*_ was set to either 0.15, 0.30 or 0.50.

**Figure 5:**
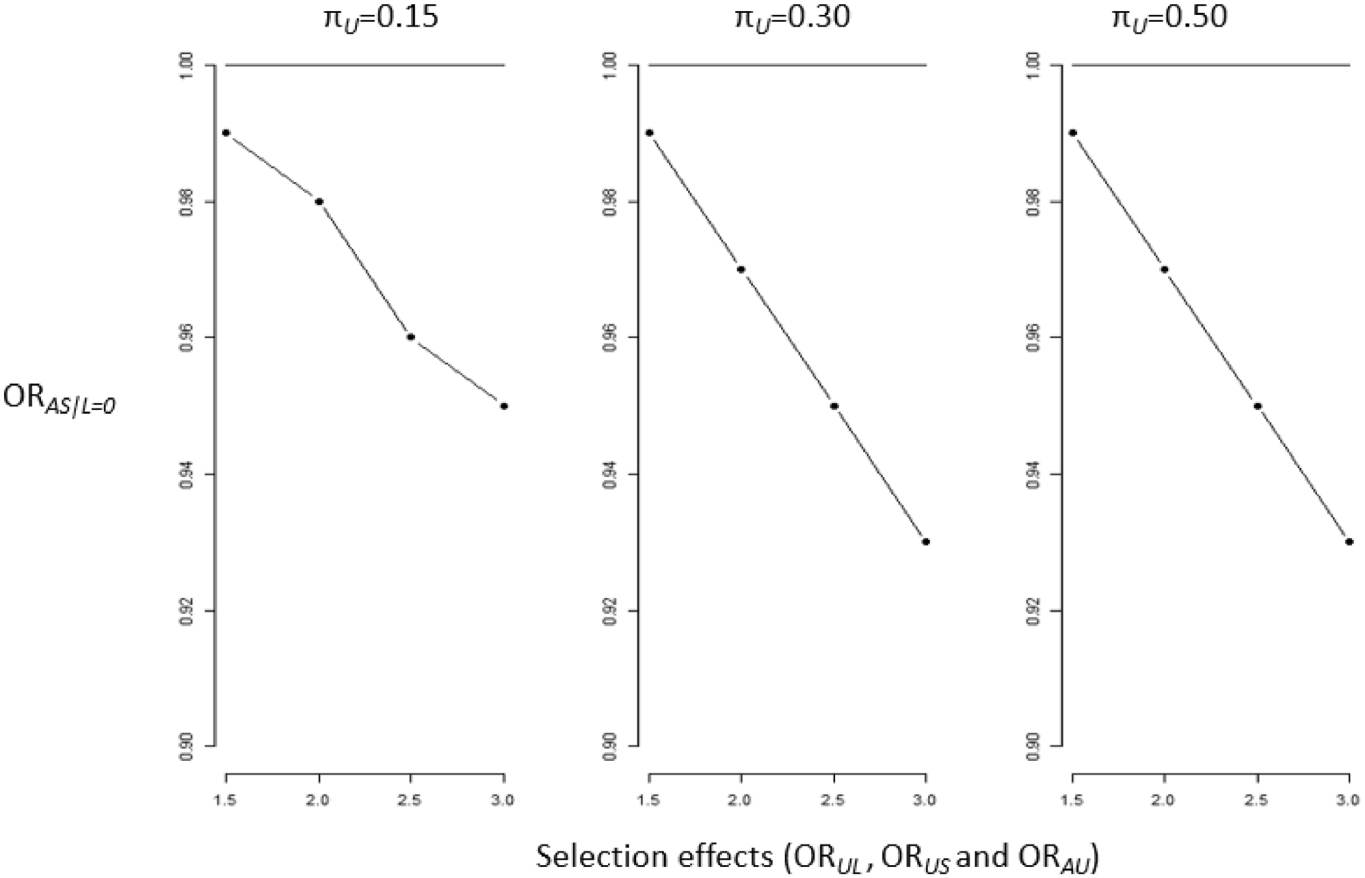
The upper straight line represents the results of the observed cohort for women aged 40 + years assuming no influence of an unmeasured confounder or selection bias. The lower lines represent the collider-stratification bias of OR_*AS|L*=*0*_ assuming a true effect of maternal age on stillbirth for women aged 40 + years without an interaction between exposure *A* and the unmeasured confounder *U.* Average odds ratio with π_L=_ 0.20 and with varying input parameters for π_*U*_ (0.15, 0.30, 0.50) and the selection effects (OR_*UL*_ and OR_*US*_). Each scenario was iterated 100 times.

In the observed cohort, the association between maternal age and stillbirth presented as a U-shape, with the lowest risk for women aged 25–29 (OR 0.98 95% CI 0.90 to 1.17). The OR_*AS*_ for women aged 35–39 years was 1.23 (95% CI 1.11 to 1.37), increasing to 1.74 (95% CI 1.42 to 2.12) for women aged 40 +. In scenario three we simulated the biased collider-stratification pathway (without interaction between the exposure *A* and the unmeasured confounder *U*) with a direct effect of the exposure *A* on the outcome *S* (with data drawn from the observed cohort). We found evidence of minimal downward bias when we compared the results from this simulation with the observed cohort in which we assumed there was no influence from unmeasured confounders nor selection bias (Table S3). Women aged 35–39 years had an OR_*AS*_ of 1.23 (95% CI 1.11 to 1.37) in the observed cohort which was only marginally higher than the average OR_*AS|L*=*0*_ of 1.21 in the simulated scenario three. The greater departure from the results of the observed cohort for women aged 35–39 years (OR_*AS|L*=*0*_ 1.18 SI 1.17 to 1.20) was evident with input parameters of π_L=_ 0.20, π_*U*_ = 0.30, OR_*UL*_ = 3.0, OR_*US*_ = 3.0. In the observed cohort, women aged 40 + years had an OR_*AS*_ of 1.74 (95% CI 1.42 to 2.12) and we found a greater departure from the observed cohort in general (Fig. 5). For example, with input parameters of parameters π_L=_ 0.20, π_*U*_ = 0.30, OR_*UL*_ = 3.0, OR_*US*_ = 3.0 the OR_*AS|L*=*0*_ for women aged 40 + years was 1.58 (SI 1.56 to 1.61).

When we simulated the true null association between exposure maternal age *A* and the outcome of stillbirth *S* (input parameters π_L=_ 0.20, π_*U*_ = 0.15, OR_*UL*_ = 1.5, OR_*US*_ = 1.5) by each maternal age in the sensitivity analysis, we found that the structure of bias was similar to when maternal age was categorised by 5-year age groups (Fig. S5).

## Discussion

Establishing the magnitude and direction of bias from unobserved early pregnancy losses on exposure-outcome associations is essential in improving our understanding of aetiological associations in perinatal epidemiology. In this simulation study, we quantified the magnitude and direction of bias due left truncation and selection in utero on the association between the exposure of advancing maternal age and the risk of stillbirth. Our findings suggest that the exclusion of early pregnancy loss in perinatal epidemiological studies likely biases effect estimates downwards. However, we found that the magnitude of bias was generally marginal, with a maximum OR_*AS|L*=*0*_ of 0.87 for women aged 40 + years when we considered a true null effect of advancing maternal age on stillbirth. The strength of this bias was primarily dependent on the selection effects of the unmeasured confounder on the collider of early pregnancy loss *L* (OR_*UL*_), the exposure of advancing maternal age *A* (OR_*AU*_) and the outcome of stillbirth *S* (OR_*US*_).

Direct comparison to other studies was constrained by differences between exposure-outcome associations and the structure of the collider-stratification bias; however, the small magnitude of bias in this study is consistent with other studies that examined the collider-stratification mechanism for other perinatal outcomes^24–32^, such as the smoking-birthweight paradox^6,24,26,27^. Our findings, and those of others, suggest that the bias resulting from a collider-stratification mechanism would need to be very strong to produce an association that reverses the observed causal effects, and that this would primarily occur in scenarios where the effect of the unmeasured confounder would be quite large. It remains uncertain as to whether it is plausible that such a large causal effect would remain unknown or unobservable. On this basis, we limited the selection effects of *U* (OR_*UL*_ and OR_*US*_) to a realistic range from 1.5 to an upper limit of 3.0. We found that the stronger the selection effects of *U* (OR_*UL*_ and OR_*US*_), the stronger the magnitude of bias regardless of the prevalence of early pregnancy *L* or the prevalence of the unmeasured confounder *U*. Simulation studies that considered an interaction between an unmeasured confounder and the exposure found evidence of a stronger magnitude of bias in comparison to simulations without an interaction effect^25,30^. Often called *depletion of susceptibles*, this interaction between the susceptible factor (in our study this would be advancing maternal age) increases the depletion of early pregnancy loss among those who experience the unmeasured confounder^33,34^. Although our study showed an increase in the magnitude of bias when we considered a depletion effect, it was only evident for women aged 40 + years. One of the benefits of this study was that we could directly compare the difference between OR_*AS|L*=*0*_ and OR_*AS*_ (the observed cohort without the simulated bias). Here, we found that the magnitude of downward bias was negligible for women aged 35–39 years and minimal for women aged 40 +. Overall, our findings indicate that the influence of bias due to left truncation and selection in utero is not sufficient to have a substantial effect on the strength of the association between advancing maternal age and stillbirth.

As simulation studies are only as valid as their assumptions, we used published literature and an observed cohort to support our assumptions of the magnitude of the underlying causal effects when quantifying the influence of bias in the association between advancing maternal age and stillbirth. Advancing maternal age has previously been established as a strong independent risk factor for early pregnancy loss in the first trimester^11^, with risks increasing incrementally after the age of 30 years. Although the absolute risk of second trimester pregnancy loss is small in comparison to first semester, there is an incremental increase for women of advancing age^35^. Using data from a 2019 Norwegian study^11^ we were able to model this incremental increase in risk of early pregnancy loss *L* prior to 20 gestational weeks for each year of maternal age from 20 to 45 years in our simulations. We accounted for a variety of early pregnancy loss scenarios from 12.8%^11^ a mid-range of 20%^19^ and an upper level of 30%^20^. As our simulations are hypothetical scenarios in which all conceptions are selected, it is also likely that induced abortions would present a small competing risk to stillbirth. However, the Norwegian study^11^, from which our lowest prevalence (12.8%) of early pregnancy loss is derived, did correct for induced abortions, finding very little difference in the overall estimate of miscarriage^11^. Although the absolute risk of stillbirth is low in high-income countries, it has not declined in recent decades despite advances in perinatal and obstetric care^14^. For women aged 40 + years, the risk of stillbirth increases earlier in pregnancy than for younger women, with a women aged 40 + having a greater risk of stillbirth at 39 gestational weeks compared to a younger women at 41 weeks^35^. Using data from our large observed cohort in Western Australia, we built models that accounted for the differential impact of the exposure advancing age *A* on the outcome of stillbirth *S* in a high-income setting. Our careful definition of our exposure variable advancing maternal age *A,* accounting for the differential impact on the early pregnancy loss *L* and stillbirth *S*, ensure our simulations are reflective of real world interactions between variables.

The exact biological mechanism of the higher risk of maternal age remains uncertain, with many of the potential shared risk factors for early pregnancy loss and stillbirth unobservable prior to the outcome. Possible suggestions include utero-placental dysfunction predisposing some women to adverse fetal outcomes including early pregnancy loss and stillbirth^36^. Infections can increase risk of early pregnancy loss and stillbirth, infecting the fetus via the placenta^37^ with many infections asymptomatic. Fetal chromosomal abnormalities are the most common cause of early pregnancy loss in the first trimester, accounting for 50% of non-recurrent pregnancy losses^38,39^. There is an increased chromosomal anomaly rate (approx. 20%) in women aged 35 + years compared to younger women in sporadic and recurrent pregnancy losses^40^. Here, chromosomal anomalies would be an ideal candidate for the unobserved variable in our second simulation scenario. Increasing advanced age predisposes mothers to increasing risk of chromosomal anomalies that increase the risk of early pregnancy loss. Notwithstanding the collider-stratification mechanism, unmeasured confounders can lead to biased exposure-outcome effect estimates in either direction. Making assumptions about such confounders that are unobservable or unknown is challenging for researchers. Given the existence of causal factors that are not measured or remain to be discovered, researchers will continue to be required to make reasonable assumptions in relation to the strength and role of such unobservable confounders in the causal pathway, as we have done in our simulation study.

Quite often, the influence of collider-stratification bias is only examined when unexpected associations are observed in epidemiological studies^24–29^. As the use of left truncated data is ubiquitous in perinatal in epidemiology, due to restriction of studies until a time when pregnancy is either observed or deemed viable, the quantification of bias should be no less important in studies when an expected association is observed. Nonetheless, there are some caveats for interpreting our simulation results. The estimates in our simulation study are based on simple scenarios with all the variables having a binary response. We further assumed that there are no other forms of bias such as misclassification, nor the effects of multiple unmeasured confounders. There may also be a mediator variable, such as a pregnancy disease, that mitigates the association between advancing maternal age and stillbirth. An additional limitation of this study on the effect of ageing on stillbirth is that we did not consider selection bias prior to conception; that is women of advancing maternal age have a higher risk of infertility^41^.

In this simulation study, we have quantified the magnitude and influence of bias from left-truncated perinatal data caused by studying cases prevalent from a specified gestation age, rather than including all cases in a conception or pregnancy cohort. We know that conditioning on the collider (early pregnancy loss prior to 20 weeks gestational weeks) will produce biased estimated in perinatal exposure-outcome associations. Using realistic assumptions, we found the magnitude of bias was generally minimal when using data that is left truncated due to early pregnancy loss on the association between the exposure of advancing maternal age and the outcome of stillbirth. When we considered a true association between the exposure and outcome, we observed a small downward bias which was stronger for women aged 40 + years. In our specific research question, in which the exposure is advancing maternal age, our findings indicated that the influence of bias due to selection in utero (and thereby left truncation) is not sufficient to have a substantial effect on the association with stillbirth. That is not to say that other researchers, with a different research question, would not find stronger evidence of bias when using left truncated birth data. However, as we demonstrated in this simulation, the strength of the bias is driven primarily by the prevalence and strength of the unmeasured confounder *U* rather than selection in utero. Although it is unlikely that such large unmeasured confounders exist, researcher should consider the influence of collider-stratification bias when using left-truncated data within the context of their own studies.

## Supplementary Information

Supplementary Information.

## Data Availability

The data that supports the findings of this study are owned by the government departments who approved the linkage and use of the data for this study. The current Human Research Ethics Committee approvals were obtained for public sharing and presentation of data on results only, meaning the unit-record level data used in this study cannot be shared by the authors. The steps involved in seeking permission for the use of the original data in this study is the same for all researchers. Researchers who wish to replicate our results can apply directly to Data Linkage, Department of Health, Western Australia. The steps to apply for data are described at https://www.datalinkage-wa.org.au.
