# Supplementary material for: Bias in the association between advanced maternal age and stillbirth using left truncated data
Source: Sci Rep. 2022 Nov 10;12:19214. doi: 10.1038/s41598-022-23719-3 (PMC9649623; doi:10.1038/s41598-022-23719-3)
Supplement: Supplementary file 1 — Supplementary Information. [file 41598_2022_23719_MOESM1_ESM.docx]

**Supplementary material**

**Bias in the association between advanced maternal age and stillbirth using left truncated data**

Jennifer Dunne, Gizachew A Tessema, Amanuel T Gebremedhin, Gavin Pereira

**Figure S1.** A directed acyclic graph (DAG) representing the effect of maternal age *A* on stillbirth *S* when conditioning on early pregnancy loss *L* with an interaction between maternal age *A* and the unmeasured confounder *U*.

**Figure S2**. A directed acyclic graph (DAG) representing the true effect of maternal age *A* on stillbirth *S* when conditioning on early pregnancy loss *L*.

**Figure S3.** Risk of stillbirth according to maternal age

**Figure S4.** Collider-stratification bias of OR*_AS|L=0_* under the true null effect of maternal age on stillbirth for women aged 35-39 years.

**Figure S5.** Average odds ratio (OR) for the association between the exposure maternal age *A* and the outcome of stillbirth *S* over 100 simulations assuming a true null effect and the input of one unmeasured confounder *U* by each maternal year

**Table S1.** Average odds ratio (OR) and 95% simulation intervals (SIs) for the association between the exposure maternal age *A* and the outcome of stillbirth *S* over 100 simulations assuming a true null effect and the input of one unmeasured confounder *U*.

**Table S2.** Average odds ratio (OR) and 95% simulation intervals (SI) for the biased association between maternal age *A* and stillbirth *S* over 100 simulations for one single unmeasured *U*, assuming a true null effect of maternal age *A* on stillbirth *S* and an interaction between *U* and the exposure of maternal age *A*.

**Table S3**. Average odds ratio (OR) and 95% simulation intervals (SIs) for the association between the exposure maternal age *A* and the outcome of stillbirth *S* over 100 simulations assuming a true effect and the input of one unmeasured confounder *U*.

**Simulation code**


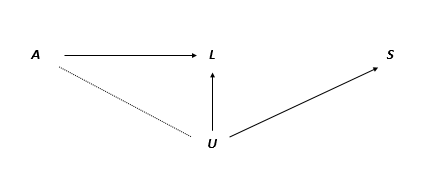


**Figure S1.** Directed acyclic graph (DAG) of the structure of collider-stratification bias with interaction between the exposure and the unmeasured confounder U. The exposure maternal age *A* affects early pregnancy loss *L*, which is also affected by the independent risk factor *U*, inducing a back-door pathway between exposure *A* and the outcome of stillbirth *S*. When there is an interaction between *A* and *U* (depicted by dashed line), there is an increase in the prevalence of early pregnancy loss L for those that are exposed to both the exposure maternal age *A* and the unmeasured confounder *U*.


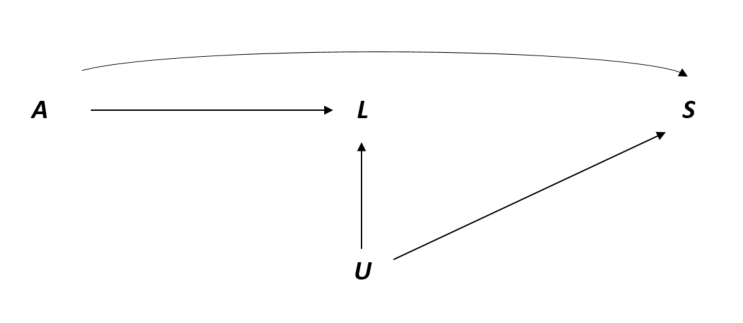


**Figure S2**. Directed acyclic graph (DAG) of the structure of collider-stratification bias. The exposure maternal age *A* affects pregnancy loss *L*, which is also affected by the independent risk factor *U*, inducing a back-door pathway between exposure *A* and the outcome of stillbirth *S*. Here, there is a true effect of maternal age *A* on the outcome of stillbirth *S*.


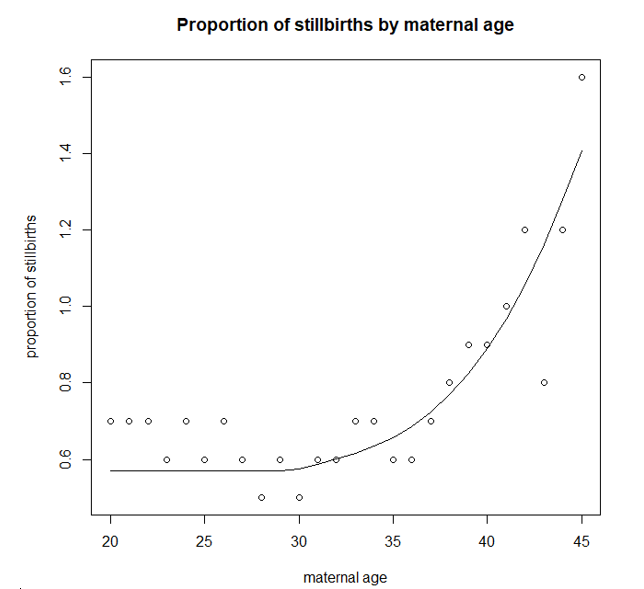


**Figure S3.** Risk of stillbirth according to maternal age based on a non-parametric regression model with locally weighted scatterplot smoothing to capture the nonlinearity of the association between maternal age and the outcome of stillbirth.


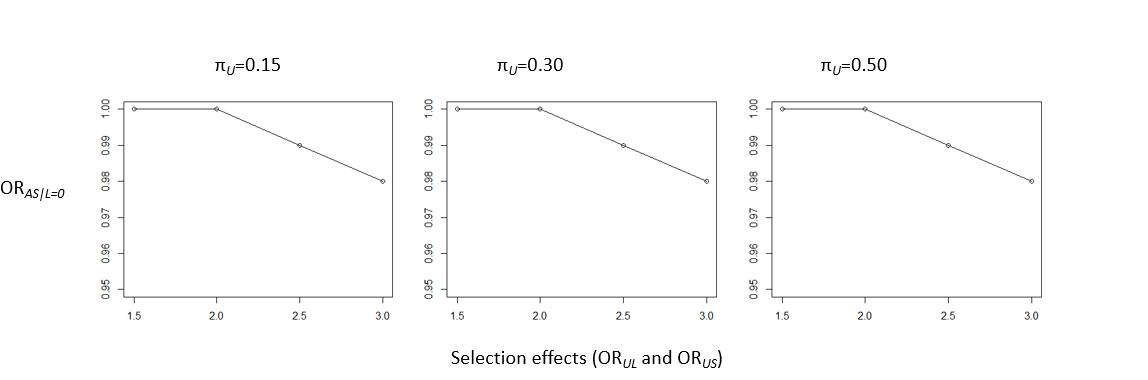


**Figure S4**. Collider-stratification bias of OR*_AS|L=0_* under the true null effect of maternal age on stillbirth for women aged 35-39 years. Average odds ratio assuming with π_L=_ 0.20 and varying input parameters for π*_U_* and the selection effects (OR*_UL_* and OR*_US_*). Each scenario was simulated 100 times.


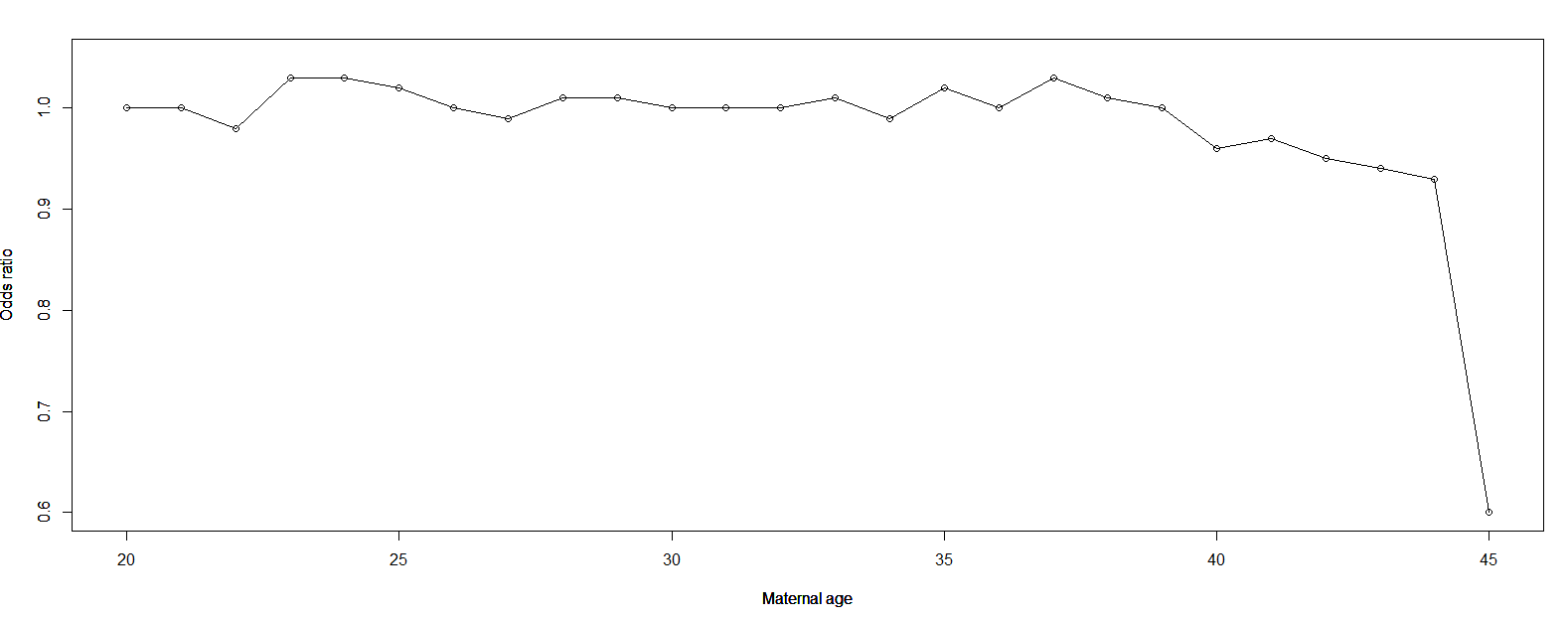


**Figure S5.** Average odds ratio (OR) for the association between the exposure maternal age *A* and the outcome of stillbirth *S* over 100 simulations assuming a true null effect the and input of one unmeasured confounder *U* by each maternal year

**Table S1.** Average odds ratio (OR) and 95% simulation intervals (SIs) for the association between the exposure maternal age *A* and the outcome of stillbirth *S* over 100 simulations assuming a true null effect and the input of one unmeasured confounder *U*.

| Selection effects | | | Average OR for maternal age on stillbirth (95% SI) | | | | |
| --- | --- | --- | --- | --- | --- | --- | --- |
| π*_L_* | π*_U_* | OR*_UL_* and OR*_US_* | 20-24 | 25-29 | 30-34 | 35-39 | 40+ |
|  |  |  |  |  |  |  |  |
| 0.128 | 0.15 | 1.5 | 1.00 (0.98 to 1.01) | 0.99 (0.99 to 1.00) | Ref | 1.00 (0.99 to 1.01) | 0.99 (0.97 to 1.01) |
|  |  | 2.0 | 1.00 (0.99 to 1.01) | 1.00 (0.99 to 1.01) | Ref | 1.00 (0.99 to 1.01) | 0.98 (0.95 to 1.00) |
|  |  | 2.5 | 1.00 (0.99 to 1.01) | 1.00 (0.99 to 1.01) | Ref | 0.99 (0.98 to 1.00) | 0.96 (0.94 to 0.98) |
|  |  | 3.0 | 1.00 (0.99 to 1.02) | 1.00 (0.99 to 1.01) | Ref | 0.98 (0.98 to 0.99) | 0.94 (0.92 to 0.96) |
|  |  |  |  |  |  |  |  |
|  | 0.30 | 1.5 | 0.99 (0.98 to 1.01) | 1.00 (0.99 to 1.00) | Ref | 1.00 (0.99 to 1.01) | 0.99 (0.96 to 1.01) |
|  |  | 2.0 | 1.00 (0.99 to 1.01) | 1.00 (0.99 to 1.01) | Ref | 1.00 (0.99 to 1.01) | 0.97 (0.95 to 0.99) |
|  |  | 2.5 | 1.00 (0.99 to 1.01) | 1.00 (0.99 to 1.01) | Ref | 0.99 (0.98 to 1.00) | 0.94 (0.92 to 0.96) |
|  |  | 3.0 | 1.00 (0.99 to 1.01) | 1.00 (1.00 to 1.01) | Ref | 0.98 (0.97 to 0.99) | 0.92 (0.90 to 0.94) |
|  |  |  |  |  |  |  |  |
|  | 0.50 | 1.5 | 1.00 (0.98 to 1.01) | 1.00 (0.99 to 1.01) | Ref | 1.00 (0.99 to 1.01) | 0.99 (0.97 to 1.01) |
|  |  | 2.0 | 1.00 (0.99 to 1.01) | 1.00 (0.99 to 1.01) | Ref | 1.00 (0.99 to 1.01) | 0.97 (0.95 to 0.99) |
|  |  | 2.5 | 1.00 (0.99 to 1.01) | 1.00 (1.00 to 1.01) | Ref | 0.99 (0.98 to 1.00) | 0.94 (0.92 to 0.96) |
|  |  | 3.0 | 1.00 (1.00 to 1.01) | 1.01 (1.00 to 1.01) | Ref | 0.98 (0.98 to 0.99) | 0.93 (0.91 to 0.94) |
|  |  |  |  |  |  |  |  |
| 0.20 | 0.15 | 1.5 | 0.99 (0.98 to 1.01) | 0.99 (0.99 to 1.00) | Ref | 1.00 (0.99 to 1.01) | 0.99 (0.96 to 1.01) |
|  |  | 2.0 | 1.00 (0.98 to 1.01) | 1.00 (0.99 to 1.00) | Ref | 1.00 (0.99 to 1.01) | 0.98 (0.96 to 1.00) |
|  |  | 2.5 | 1.00 (0.99 to 1.01) | 1.00 (0.99 to 1.01) | Ref | 0.99 (0.98 to 1.00) | 0.96 (0.94 to 0.99) |
|  |  | 3.0 | 1.00 (0.99 to 1.01) | 1.00 (0.99 to 1.01) | Ref | 0.98 (0.97 to 0.99) | 0.95 (0.93 to 0.97) |
|  |  |  |  |  |  |  |  |
|  | 0.30 | 1.5 | 0.99 (0.98 to 1.01) | 1.00 (0.99 to 1.00) | Ref | 1.00 (0.99 to 1.01) | 0.99 (0.96 to 1.01) |
|  |  | 2.0 | 1.00 (0.98 to 1.01) | 1.00 (0.99 to 1.01) | Ref | 1.00 (0.99 to 1.01) | 0.97 (0.94 to 0.99) |
|  |  | 2.5 | 1.00 (0.99 to 1.01) | 1.00 (0.99 to 1.01) | Ref | 0.99 (0.98 to 1.00) | 0.95 (0.92 to 0.97) |
|  |  | 3.0 | 1.00 (0.99 to 1.01) | 1.00 (1.00 to 1.01) | Ref | 0.98 (0.97 to 0.99) | 0.93 (0.91 to 0.95) |
|  |  |  |  |  |  |  |  |
|  | 0.50 | 1.5 | 0.99 (0.98 to 1.01) | 1.00 (0.99 to 1.01) | Ref | 1.00 (0.99 to 1.01) | 0.99 (0.97 to 1.01) |
|  |  | 2.0 | 1.00 (0.99 to 1.01) | 1.00 (0.99 to 1.01) | Ref | 1.00 (0.99 to 1.01) | 0.97 (0.95 to 0.99) |
|  |  | 2.5 | 1.00 (0.99 to 1.01) | 1.00 (1.00 to 1.01) | Ref | 0.99 (0.98 to 1.00) | 0.95 (0.93 to 0.98) |
|  |  | 3.0 | 1.00 (0.99 to 1.01) | 1.01 (1.00 to 1.01) | Ref | 0.98 (0.97 to 0.99) | 0.93 (0.91 to 0.95) |
|  |  |  |  |  |  |  |  |
| 0.30 | 0.15 | 1.5 | 1.00 (0.98 to 1.01) | 0.99 (0.99 to 1.00) | Ref | 1.00 (0.99 to 1.02) | 0.99 (0.96 to 1.02) |
|  |  | 2.0 | 1.00 (0.98 to 1.01) | 1.00 (0.99 to 1.01) | Ref | 1.00 (0.99 to 1.01) | 0.98 (0.95 to 1.01) |
|  |  | 2.5 | 1.00 (0.99 to 1.01) | 1.00 (0.99 to 1.01) | Ref | 0.99 (0.98 to 1.01) | 0.96 (0.94 to 0.99) |
|  |  | 3.0 | 1.00 (0.99 to 1.01) | 1.00 (0.99 to 1.01) | Ref | 0.99 (0.98 to 1.00) | 0.95 (0.93 to 0.98) |
|  |  |  |  |  |  |  |  |
|  | 0.30 | 1.5 | 0.99 (0.98 to 1.01) | 1.00 (0.99 to 1.01) | Ref | 1.00 (0.99 to 1.01) | 0.99 (0.96 to 1.02) |
|  |  | 2.0 | 1.00 (0.98 to 1.01) | 1.00 (0.99 to 1.01) | Ref | 1.00 (0.99 to 1.01) | 0.98 (0.75 to 1.26) |
|  |  | 2.5 | 1.00 (0.99 to 1.01) | 1.00 (1.00 to 1.01) | Ref | 0.99 (0.98 to 1.00) | 0.95 (0.92 to 0.98) |
|  |  | 3.0 | 1.00 (0.99 to 1.01) | 1.00 (1.00 to 1.01) | Ref | 0.98 (0.97 to 0.99) | 0.94 (0.91 to 0.96) |
|  |  |  |  |  |  |  |  |
|  | 0.50 | 1.5 | 1.00 (0.99 to 1.01) | 1.00 (0.99 to 1.01) | Ref | 1.00 (0.99 to 1.02) | 1.00 (0.97 to 1.03) |
|  |  | 2.0 | 1.00 (0.99 to 1.01) | 1.00 (1.00 to 1.01) | Ref | 1.00 (0.99 to 1.01) | 0.99 (0.96 to 1.01) |
|  |  | 2.5 | 1.00 (0.99 to 1.01) | 1.01 (1.00 to 1.01) | Ref | 0.99 (0.98 to 1.00) | 0.96 (0.94 to 0.99) |
|  |  | 3.0 | 1.00 (0.99 to 1.01) | 1.00 (1.00 to 1.01) | Ref | 0.98 (0.97 to 0.98) | 0.94 (0.92 to 0.96) |

OR*_AS|L=0_* odds ratio for the association between the exposure maternal age *A* and the outcome stillbirth *S* when early pregnancy loss *L* is set to 0; SI simulation intervals; π*_L_* early pregnancy loss; π*_U_* unmeasured confounder; OR*_UL_* odds ratio for the association between the unmeasured confounder *U* and early pregnancy loss *L*; OR*_US_* odds ratio for the association between the unmeasured confounder *U* and stillbirth *S*

**Table S2.** Average odds ratio (OR) and 95% simulation intervals (SI) for the biased association between maternal age *A* and stillbirth *S* over 100 simulations for one single unmeasured *U*, assuming a true null effect of maternal age *A* on stillbirth *S* and an interaction between *U* and the exposure of maternal age *A*.

| Selection effects | | | Average OR for maternal age on stillbirth (95% SI) | | | | |
| --- | --- | --- | --- | --- | --- | --- | --- |
| π*_L_* | π*_U_* | OR*_UL,_* OR*_US_* OR*_AU_* | 20-24 | 25-29 | 30-34 | 35-39 | 40+ |
|  |  |  |  |  |  |  |  |
| 0.128 | 0.15 | 1.5 | 0.99 (0.98 to 1.01) | 0.99 (0.99 to 1.00) | Ref | 1.00 (0.99 to 1.01) | 0.98 (0.96 to 1.01) |
|  |  | 2.0 | 0.99 (0.98 to 1.01) | 1.00 (0.99 to 1.01) | Ref | 1.00 (0.99 to 1.01) | 0.98 (0.96 to 1.00) |
|  |  | 2.5 | 0.99 (0.98 to 1.00) | 0.99 (0.99 to 1.00) | Ref | 1.00 (0.99 to 1.01) | 0.97 (0.95 to 0.99) |
|  |  | 3.0 | 0.99 (0.98 to 1.00) | 0.99 (0.99 to 1.00) | Ref | 1.00 (0.99 to 1.01) | 0.96 (0.93 to 0.97) |
|  |  |  |  |  |  |  |  |
|  | 0.30 | 1.5 | 0.99 (0.98 to 1.01) | 1.00 (0.99 to 1.00) | Ref | 1.00 (0.99 to 1.01) | 0.99 (0.97 to 1.02) |
|  |  | 2.0 | 0.99 (0.98 to 1.01) | 1.00 (0.99 to 1.01) | Ref | 1.00 (0.99 to 1.01) | 0.98 (0.96 to 1.00) |
|  |  | 2.5 | 0.99 (0.98 to 1.00) | 1.00 (0.99 to 1.00) | Ref | 1.00 (1.00 to 1.01) | 0.96 (0.94 to 0.98) |
|  |  | 3.0 | 0.99 (0.98 to 1.00) | 1.00 (0.99 to 1.00) | Ref | 1.00 (0.99 to 1.01) | 0.95 (0.93 to 0.97) |
|  |  |  |  |  |  |  |  |
|  | 0.50 | 1.5 | 0.99 (0.98 to 1.01) | 1.00 (0.99 to 1.00) | Ref | 1.00 (0.99 to 1.01) | 1.00 (0.98 to 1.02) |
|  |  | 2.0 | 0.99 (0.98 to 1.01) | 1.00 (0.99 to 1.01) | Ref | 1.00 (0.99 to 1.01) | 0.98 (0.96 to 1.00) |
|  |  | 2.5 | 0.99 (0.98 to 1.00) | 0.99 (0.99 to 1.00) | Ref | 1.00 (1.00 to 1.01) | 0.97 (0.95 to 0.99) |
|  |  | 3.0 | 0.99 (0.98 to 1.00) | 0.99 (0.99 to 1.00) | Ref | 1.00 (0.99 to 1.01) | 0.95 (0.93 to 0.97) |
|  |  |  |  |  |  |  |  |
| 0.20 | 0.15 | 1.5 | 0.99 (0.98 to 1.01) | 0.99 (0.99 to 1.00) | Ref | 1.00 (0.99 to 1.01) | 0.99 (0.97 to 1.01) |
|  |  | 2.0 | 0.99 (0.98 to 1.01) | 1.00 (0.99 to 1.00) | Ref | 1.00 (0.99 to 1.01) | 0.98 (0.95 to 1.00) |
|  |  | 2.5 | 0.99 (0.98 to 1.01) | 1.00 (0.99 to 1.00) | Ref | 1.00 (0.99 to 1.01) | 0.96 (0.93 to 0.98) |
|  |  | 3.0 | 0.99 (0.98 to 1.01) | 1.00 (0.99 to 1.00) | Ref | 0.99 (0.98 to 1.00) | 0.94 (0.92 to 0.96) |
|  |  |  |  |  |  |  |  |
|  | 0.30 | 1.5 | 0.99 (0.98 to 1.00) | 1.00 (0.99 to 1.00) | Ref | 1.00 (0.99 to 1.01) | 0.99 (0.96 to 1.01) |
|  |  | 2.0 | 0.99 (0.98 to 1.00) | 1.00 (0.99 to 1.00) | Ref | 1.00 (0.99 to 1.01) | 0.97 (0.95 to 0.99) |
|  |  | 2.5 | 0.99 (0.98 to 1.00) | 1.00 (0.99 to 1.00) | Ref | 1.00 (0.99 to 1.01) | 0.94 (0.92 to 0.96) |
|  |  | 3.0 | 0.99 (0.98 to 1.00) | 1.00 (0.99 to 1.00) | Ref | 0.99 (0.98 to 1.00) | 0.92 (0.90 to 0.94) |
|  |  |  |  |  |  |  |  |
|  | 0.50 | 1.5 | 0.99 (0.98 to 1.00) | 1.00 (0.99 to 1.00) | Ref | 1.00 (0.99 to 1.01) | 0.99 (0.96 to 1.01) |
|  |  | 2.0 | 0.99 (0.98 to 1.01) | 1.00 (0.99 to 1.00) | Ref | 1.00 (0.99 to 1.01) | 0.97 (0.95 to 0.99) |
|  |  | 2.5 | 0.99 (0.98 to 1.00) | 1.00 (0.99 to 1.00) | Ref | 1.00 (0.99 to 1.01) | 0.94 (0.92 to 0.96) |
|  |  | 3.0 | 0.99 (0.98 to 1.00) | 1.00 (0.99 to 1.00) | Ref | 0.99 (0.98 to 1.00) | 0.92 (0.90 to 0.94) |
|  |  |  |  |  |  |  |  |
| 0.30 | 0.15 | 1.5 | 0.99 (0.98 to 1.01) | 1.00 (0.99 to 1.00) | Ref | 1.00 (0.99 to 1.01) | 0.98 (0.95 to 1.01) |
|  |  | 2.0 | 1.00 (0.99 to 1.01) | 1.00 (0.99 to 1.01) | Ref | 1.00 (0.99 to 1.01) | 0.96 (0.93 to 0.99) |
|  |  | 2.5 | 1.00 (0.99 to 1.01) | 1.00 (0.99 to 1.01) | Ref | 0.99 (0.98 to 1.00) | 0.93 (0.90 to 0.96) |
|  |  | 3.0 | 1.00 (0.99 to 1.01) | 1.00 (0.99 to 1.01) | Ref | 0.98 (0.97 to 0.99) | 0.90 (0.88 to 0.92) |
|  |  |  |  |  |  |  |  |
|  | 0.30 | 1.5 | 0.99 (0.98 to 1.01) | 1.00 (0.99 to 1.00) | Ref | 1.00 (0.99 to 1.01) | 0.98 (0.95 to 1.00) |
|  |  | 2.0 | 1.00 (0.99 to 1.01) | 1.00 (0.99 to 1.01) | Ref | 1.00 (0.99 to 1.01) | 0.94 (0.92 to 0.97) |
|  |  | 2.5 | 1.00 (0.98 to 1.01) | 1.00 (0.99 to 1.01) | Ref | 0.99 (0.98 to 1.00) | 0.91 (0.88 to 0.93) |
|  |  | 3.0 | 1.00 (0.99 to 1.01) | 1.00 (0.99 to 1.01) | Ref | 0.98 (0.97 to 0.99) | 0.87 (0.84 to 0.89) |
|  |  |  |  |  |  |  |  |
|  | 0.50 | 1.5 | 0.99 (0.98 to 1.01) | 1.00 (0.99 to 1.01) | Ref | 1.00 (0.99 to 1.01) | 0.98 (0.95 to 1.00) |
|  |  | 2.0 | 1.00 (0.99 to 1.01) | 1.00 (0.99 to 1.01) | Ref | 1.00 (0.99 to 1.01) | 0.95 (0.92 to 0.97) |
|  |  | 2.5 | 1.00 (0.99 to 1.01) | 1.01 (1.00 to 1.01) | Ref | 0.99 (0.98 to 1.00) | 0.92 (0.89 to 0.94) |
|  |  | 3.0 | 1.00 (0.99 to 1.01) | 1.00 (1.00 to 1.01) | Ref | 0.98 (0.98 to 0.99) | 0.88 (0.86 to 0.90) |

OR*_AS|L=0_* odds ratio for the association between the exposure maternal age *A* and the outcome stillbirth *S* when early pregnancy loss *L* is set to 0; SI simulation intervals; π*_L_* early pregnancy loss; π*_U_* unmeasured confounder; OR*_UL_* odds ratio for the association between the unmeasured confounder *U* and early pregnancy loss *L*; OR*_US_* odds ratio for the association between the unmeasured confounder *U* and stillbirth *S*; OR*_AU_* odds ratio for the association between the advanced maternal age *A* and the unmeasured confounder *U*

**Table S3**. Average odds ratio (OR) and 95% simulation intervals (SIs) for the association between the exposure maternal age *A* and the outcome of stillbirth *S* over 100 simulations assuming a true effect and the input of one unmeasured confounder *U*.

| Selection effects | | | Average OR for maternal age on stillbirth (95% SI) | | | | |
| --- | --- | --- | --- | --- | --- | --- | --- |
|  | | | 20-24 | 25-29 | 30-34 | 35-39 | 40+ |
|  | | |  |  |  |  |  |
| Original Cohort (OR 95% CI) | | | 1.16 (1.05 to 1.29) | 0.98 (0.90 to 1.17) | Ref | 1.23 (1.11 to 1.37) | 1.74 (1.42 to 2.12) |
|  |  |  |  |  |  |  |  |
| π*_L_* | π*_U_* | OR*_UL_* and OR*_US_* | 20-24 | 25-29 | 30-34 | 35-39 | 40+ |
|  |  |  |  |  |  |  |  |
| 0.128 | 0.15 | 1.5 | 0.94 (0.93 to 0.95) | 0.94 (0.93 to 0.95) | Ref | 1.21 (1.20 to 1.23) | 1.71 (1.67 to 1.74) |
|  |  | 2.0 | 0.94 (0.93 to 0.95) | 0.94 (0.94 to 0.95) | Ref | 1.21 (1.22 to 1.35) | 1.69 (1.66 to 1.71) |
|  |  | 2.5 | 0.95 (0.93 to 0.96) | 0.95 (0.94 to 0.95) | Ref | 1.20 (1.19 to 1.22) | 1.67 (1.64 to 1.69) |
|  |  | 3.0 | 0.95 (0.94 to 0.96) | 0.95 (0.94 to 0.96) | Ref | 1.19 (1.18 to 1.21) | 1.62 (1.60 to 1.65) |
|  |  |  |  |  |  |  |  |
|  | 0.30 | 1.5 | 0.94 (0.93 to 0.95) | 0.94 (0.94 to 0.95) | Ref | 1.21 (1.20 to 1.22) | 1.69 (1.66 to 1.72) |
|  |  | 2.0 | 0.94 (0.93 to 0.95) | 0.95 (0.94 to 0.95) | Ref | 1.21 (1.19 to 1.22) | 1.67 (1.64 to 1.69) |
|  |  | 2.5 | 0.94 (0.93 to 0.95) | 0.95 (0.94 to 0.96) | Ref | 1.20 (1.19 to 1.21) | 1.62 (1.60 to 1.65) |
|  |  | 3.0 | 0.95 (0.94 to 0.96) | 0.95 (0.94 to 0.96) | Ref | 1.19 (1.18 to 1.20) | 1.58 (1.55 to 1.60) |
|  |  |  |  |  |  |  |  |
|  | 0.50 | 1.5 | 0.94 (0.93 to 0.95) | 0.94 (0.94 to 0.95) | Ref | 1.21 (1.20 to 1.23) | 1.67 (1.65 to 1.71) |
|  |  | 2.0 | 0.94 (0.93 to 0.95) | 0.95 (0.94 to 0.96) | Ref | 1.21 (1.20 to 1.22) | 1.66 (1.64 to 1.69) |
|  |  | 2.5 | 0.95 (0.94 to 0.96) | 0.95 (0.95 to 0.96) | Ref | 1.20 (1.19 to 1.21) | 1.61 (1.59 to 1.63) |
|  |  | 3.0 | 0.95 (0.94 to 0.96) | 0.95 (0.95 to 0.96) | Ref | 1.19 (1.18 to 1.20) | 1.57 (1.55 to 1.59) |
|  |  |  |  |  |  |  |  |
| 0.20 | 0.15 | 1.5 | 0.94 (0.93 to 0.95) | 0.94 (0.93 to 0.95) | Ref | 1.21 (1.20 to 1.23) | 1.70 (1.67 to 1.74) |
|  |  | 2.0 | 0.94 (0.93 to 0.95) | 0.94 (0.93 to 0.95) | Ref | 1.21 (1.19 to 1.22) | 1.69 (1.66 to 1.72) |
|  |  | 2.5 | 0.94 (0.93 to 0.96) | 0.95 (0.94 to 0.95) | Ref | 1.20 (1.19 to 1.21) | 1.66 (1.63 to 1.69) |
|  |  | 3.0 | 0.95 (0.93 to 0.96) | 0.95 (0.94 to 0.95) | Ref | 1.19 (1.18 to 1.20) | 1.63 (1.60 to 1.66) |
|  |  |  |  |  |  |  |  |
|  | 0.30 | 1.5 | 0.94 (0.92 to 0.95) | 0.94 (0.94 to 0.95) | Ref | 1.21 (1.20 to 1.22) | 1.69 (1.66 to 1.72) |
|  |  | 2.0 | 0.94 (0.93 to 0.95) | 0.95 (0.94 to 0.95) | Ref | 1.21 (1.19 to 1.22) | 1.66 (1.63 to 1.69) |
|  |  | 2.5 | 0.94 (0.93 to 0.95) | 0.95 (0.94 to 0.96) | Ref | 1.20 (1.19 to 1.21) | 1.62 (1.60 to 1.65) |
|  |  | 3.0 | 0.95 (0.93 to 0.96) | 0.95 (0.94 to 0.96) | Ref | 1.18 (1.17 to 1.20) | 1.58 (1.56 to 1.61) |
|  |  |  |  |  |  |  |  |
|  | 0.50 | 1.5 | 0.94 (0.93 to 0.95) | 0.94 (0.94 to 0.95) | Ref | 1.21 (1.20 to 1.23) | 1.69 (1.66 to 1.72) |
|  |  | 2.0 | 0.94 (0.93 to 0.95) | 0.95 (0.94 to 0.96) | Ref | 1.21 (1.20 to 1.22) | 1.65 (1.62 to 1.68) |
|  |  | 2.5 | 0.95 (0.94 to 0.96) | 0.95 (0.94 to 0.96) | Ref | 1.20 (1.19 to 1.21) | 1.62 (1.60 to 1.64) |
|  |  | 3.0 | 0.95 (0.94 to 0.96) | 0.95 (0.95 to 0.96) | Ref | 1.19 (1.18 to 1.20) | 1.58 (1.56 to 1.61) |
|  |  |  |  |  |  |  |  |
| 0.30 | 0.15 | 1.5 | 0.94 (0.93 to 0.96) | 0.94 (0.93 to 0.95) | Ref | 1.21 (1.20 to 1.23) | 1.69 (1.66 to 1.73) |
|  |  | 2.0 | 0.94 (0.93 to 0.96) | 0.94 (0.94 to 0.95) | Ref | 1.21 (1.19 to 1.22) | 1.67 (1.64 to 1.71) |
|  |  | 2.5 | 0.94 (0.93 to 0.96) | 0.95 (0.94 to 0.96) | Ref | 1.20 (1.19 to 1.22) | 1.66 (1.62 to 1.70) |
|  |  | 3.0 | 0.95 (0.93 to 0.96) | 0.95 (0.94 to 0.95) | Ref | 1.20 (1.18 to 1.21) | 1.63 (1.60 to 1.67) |
|  |  |  |  |  |  |  |  |
|  | 0.30 | 1.5 | 0.94 (0.92 to 0.95) | 0.94 (0.94 to 0.95) | Ref | 1.21 (1.20 to 1.23) | 1.68 (1.65 to 1.72) |
|  |  | 2.0 | 0.94 (0.93 to 0.95) | 0.95 (0.94 to 0.96) | Ref | 1.20 (1.19 to 1.22) | 1.66 (1.63 to 1.69) |
|  |  | 2.5 | 0.94 (0.93 to 0.95) | 0.95 (0.94 to 0.96) | Ref | 1.19 (1.18 to 1.21) | 1.63 (1.60 to 1.66) |
|  |  | 3.0 | 0.94 (0.93 to 0.95) | 0.95 (0.94 to 0.96) | Ref | 1.18 (1.17 to 1.20) | 1.59 (1.56 to 1.63) |
|  |  |  |  |  |  |  |  |
|  | 0.50 | 1.5 | 0.94 (0.93 to 0.95) | 0.95 (0.94 to 0.95) | Ref | 1.21 (1.20 to 1.23) | 1.68 (1.64 to 1.71) |
|  |  | 2.0 | 0.95 (0.93 to 0.96) | 0.95 (0.94 to 0.96) | Ref | 1.21 (1.19 to 1.22) | 1.67 (1.63 to 1.70) |
|  |  | 2.5 | 0.95 (0.93 to 0.96) | 0.96 (0.95 to 0.96) | Ref | 1.20 (1.18 to 1.21) | 1.63 (1.60 to 1.66) |
|  |  | 3.0 | 0.95 (0.93 to 0.96) | 0.95 (0.94 to 0.96) | Ref | 1.18 (1.17 to 1.19) | 1.59 (1.55 to 1.62) |

OR*_AS_* odds ratio for the association between the advanced maternal age *A* and the outcome of stillbirth *S*; SI simulation intervals; π*_L_* early pregnancy loss; π*_U_* unmeasured confounder; OR*_UL_* odds ratio for the association between the unmeasured confounder *U* and early pregnancy loss *L*; OR*_US_* odds ratio for the association between the unmeasured confounder *U* and stillbirth *S*; OR*_AS_* odds ratio for the association between the advanced maternal age *A* and the outcome of stillbirth *S*

**Simulation code**

age->early pregnancy loss<-U->stillbirth

n sample size

p prevalence of U

min.bpl baseline risk of early pregnancy loss (derived from Figure 1)

or1 odds ratio for the U-early pregnancy loss effect

bY baseline odds of exposure (derived from original cohort)

or2 odds ratio for the U-stillbirth effect

Results = foreach ( i=1:100, .packages = c("MASS","sandwich","lmtest","tidyverse","Rlab","dplyr","matrixStats"), .combine=rbind) %dopar% {

rboundednorm <- function(n, mymean, mysd, min = 20, max = 45) {

a = pnorm(c(min, max), mymean, mysd)

z = runif(n, a[1], a[2])

qnorm(z, mymean, mysd)}

n=500000;p=0.5;min.bpL=9.25;or1=1.5;bY=0.007;or2=1.5

set.seed(i)

bias <- data.frame("id" = 1:n) %>%

mutate(age = rboundednorm(n, mymean=age.mean_std, mysd=age.sd_std),

bpL = (min.bpL + age.to.misc(agevec=age,min.age=x2.2[p2.2==min(p2.2)],min.risk=min(p2.2)))/100,

bL = bpL / (1 - bpL),

U = rbern(n,p),

prob_loss = plogis(log(bL) + log(or1)*U),

loss = rbern(n, prob_loss),

pY = plogis(log(bY) + log(or2)*U),

Y = rbern(n, pY)) %>%

mutate(age_cat = cut(age,breaks=c(10, 20, 24, 29, 35, 40, Inf),

labels=c("<20", "20-24", "25-29", "30-34", "35-39", "40+"), include.lowest=TRUE),

age_cat = relevel(age_cat, ref ="30-34"))#30-34 years set as reference

# fit a logistic model among live births

log_model <- bias %>% glm(formula = Y ~ age_cat,family = binomial(link = "logit"),

data = ., subset = loss==0)

ct=coeftest(log_model, vcov = sandwich)

ci=confint(ct)

c(ct[-1,1],ci[-1,1],ci[-1,2])

}
